# Chemotherapy resistance and metastasis-promoting effects of thyroid hormone in hepatocarcinoma cells are mediated by suppression of FoxO1 and Bim pathway

**DOI:** 10.1038/cddis.2016.227

**Published:** 2016-08-04

**Authors:** Hsiang-Cheng Chi, Shen-Liang Chen, Yi-Hung Cheng, Tzu-Kang Lin, Chung-Ying Tsai, Ming-Ming Tsai, Yang-Hsiang Lin, Ya-Hui Huang, Kwang-Huei Lin

**Affiliations:** 1 Department of Biochemistry, College of Medicine, Chang-Gung University, Taoyuan 333, Taiwan; 2 Department of Life Sciences, National Central University, Taoyuan 333, Taiwan, Republic of China; 3 Division of Neurosurgery, Chang Gung Memorial Hospital Linkou and Chang Gung University, Taoyuan 333, Taiwan, Republic of China; 4 Department of Nursing, Chang-Gung University of Science and Technology, Taoyuan 333, Taiwan; 5 Department of General Surgery, Chang Gung Memorial Hospital Chiayi 613, Taiwan; 6 Liver Research Center, Department of Hepato-Gastroenterology, Chang Gung Memorial Hospital, Linkou, Taoyuan 333, Taiwan

## Abstract

Hepatocellular carcinoma (HCC) is the third leading cause of cancer-related death worldwide, and systemic chemotherapy is the major treatment strategy for late-stage HCC patients. Poor prognosis following chemotherapy is the general outcome owing to recurrent resistance. Recent studies have suggested that in addition to cytotoxic effects on tumor cells, chemotherapy can induce an alternative cascade that supports tumor growth and metastasis. In the present investigation, we showed that thyroid hormone (TH), a potent hormone-mediating cellular differentiation and metabolism, acts as an antiapoptosis factor upon challenge of thyroid hormone receptor (TR)-expressing HCC cells with cancer therapy drugs, including cisplatin, doxorubicin and tumor necrosis factor-related apoptosis-inducing ligand (TRAIL). TH/TR signaling promoted chemotherapy resistance through negatively regulating the pro-apoptotic protein, Bim, resulting in doxorubicin-induced metastasis of chemotherapy-resistant HCC cells. Ectopic expression of *Bim* in hepatoma cells challenged with chemotherapeutic drugs abolished TH/TR-triggered apoptosis resistance and metastasis. Furthermore, *Bim* expression was directly transactivated by Forkhead box protein O1 (FoxO1), which was negatively regulated by TH/TR. TH/TR suppressed FoxO1 activity through both transcriptional downregulation and nuclear exclusion of FoxO1 triggered by Akt-mediated phosphorylation. Ectopic expression of the constitutively active FoxO1 mutant, FoxO1-AAA, but not FoxO1-wt, diminished the suppressive effect of TH/TR on *Bim*. Our findings collectively suggest that expression of *Bim* is mediated by FoxO1 and indirectly downregulated by TH/TR, leading to chemotherapy resistance and doxorubicin-promoted metastasis of hepatoma cells.

Hormones are molecules produced by glands in the body that enter the bloodstream and influence the behavior of another group of cells located distally. Aberrant hormone levels are implicated in the formation of several cancers. For instance, excessive estrogen or progesterone is reported to promote cellular growth of breast and prostate tumors, with antiestrogen and progesterone currently used as the main treatment strategies for these cancer types.^1, 2^ Thyroid hormones (TH), mainly 3,3′-5-tri-iodo-L-thyronine (T_3_), are potent mediators of multiple physiological activities, including cellular differentiation, metabolic rate, digestive functions and lipid metabolism.^3, 4^ The actions of T_3_ are executed via binding to thyroid hormone receptor (TR) located in the nucleus. TRs are ligand-dependent transcription factors encoded by two genes, *TRα* and *TRβ*. Upon binding of T_3_, TRs release associated co-repressors and recruit transcriptional co-activators to initiate target gene transcription.^5^ Liver is one of the major target organs of T_3_, and body TH levels are closely correlated with multiple liver-associated diseases, such as hepatocellular carcinoma (HCC).^6, 7, 8, 9^

In the diethylnitrosamine (DEN)-induced HCC animal model,^10, 11^ T_3_ has been identified as a potent inhibitor of HCC development, although significant associated mitogenic effects on hepatocytes are reported. Conversely, in chemotherapy-resistant populations of advanced HCC cells, TH/TR has been shown to promote tumor cell metastasis via upregulation of several extracellular matrix (ECM) proteases, such as matrix metalloproteinase (MMP)-2, -7 and -9.^12^

The observed effects of TH/TR on HCC development signify hormone-mediated targeting of pathways leading to apoptosis to achieve stage-specific outcomes. The conserved intrinsic mitochondrial apoptosis pathway is controlled by interplay between subgroups of the Bcl-2 family,^13^ specifically, pro-apoptotic multi-domain, antiapoptotic and pro-apoptotic Bcl-2-homology domain 3 only (BH3-only) proteins. The members of the BH3-only subfamily of proteins share only the short BH3 domain with members of the Bcl-2 family. The expression patterns and activities of BH3-only proteins are strictly regulated through complex cellular signals at both the transcriptional and posttranscriptional levels. These proteins have significant roles in initiating various physiological apoptotic events, including developmentally programmed cell death and stress-associated apoptosis.^13, 14^

Bcl-2-interacting mediator of cell death (Bim) is one of the BH3-only proteins that exhibits potent ability to bind all anti-apoptotic Bcl-2 proteins with high affinity to trigger cell death.^15^ In addition to its intrinsic toxicity, alternative splicing generates a variety of Bim isoforms with different cellular toxicities and modes of regulation.^16, 17, 18^ Among these, Bim-_EL_, Bim-_L_ and Bim-_S,_ differing in proapoptotic activity, have been most extensively studied. In view of the finding that suppression of Bim promotes metastasis and chemoresistance of tumor cells, considerable research attention has focused on its utility as an inducer of cell death and thus a potential target for tumor therapy.^19, 20, 21^

Expression of *Bim* is mainly controlled by transcription factors of the class O (FoxO) of the Forkhead box-containing protein family.^22, 23^ FoxO transcription factors, including FoxO1 (FKHR), FoxO3 (FKHRL1), FoxO4 (AFX) and FoxO6, bind DNA through a conserved forkhead box. They are additionally recruited to target sites through protein–protein interactions with other transcription factors, such as nuclear receptors,^24^ and are critical for various cellular physiological functions, including cell cycle regulation and apoptosis.^25, 26, 27^ Transactivational activity of these proteins on target genes is highly dependent on their subcellular localization. For instance, in cells where the PI3k-Akt pathway is activated, FoxO1 is phosphorylated at residues T24, S256 and S319 and later shuttled out of nucleus, resulting in loss of binding to target regulatory elements.^28^ Mutation of these three residues to alanine creates a constitutively active mutant (FoxO1-AAA), which cannot be phosphorylated by PI3k-Akt pathway and therefore remains in the nucleus.

In the current study, we showed that expression of *Bim* is mediated by FoxO1 and indirectly downregulated by TH/TR. T_3_/TR suppresses FoxO1 through transcriptional regulation and Akt-mediated nuclear exclusion and degradation. These events subsequently lead to chemotherapy resistance and doxorubicin-promoted metastasis of TR-expressing hepatoma cells.

## Results

### T_3_ regulates *Bim* mRNA and protein levels in TR-overexpressing hepatoma cells

**Figure 1:**
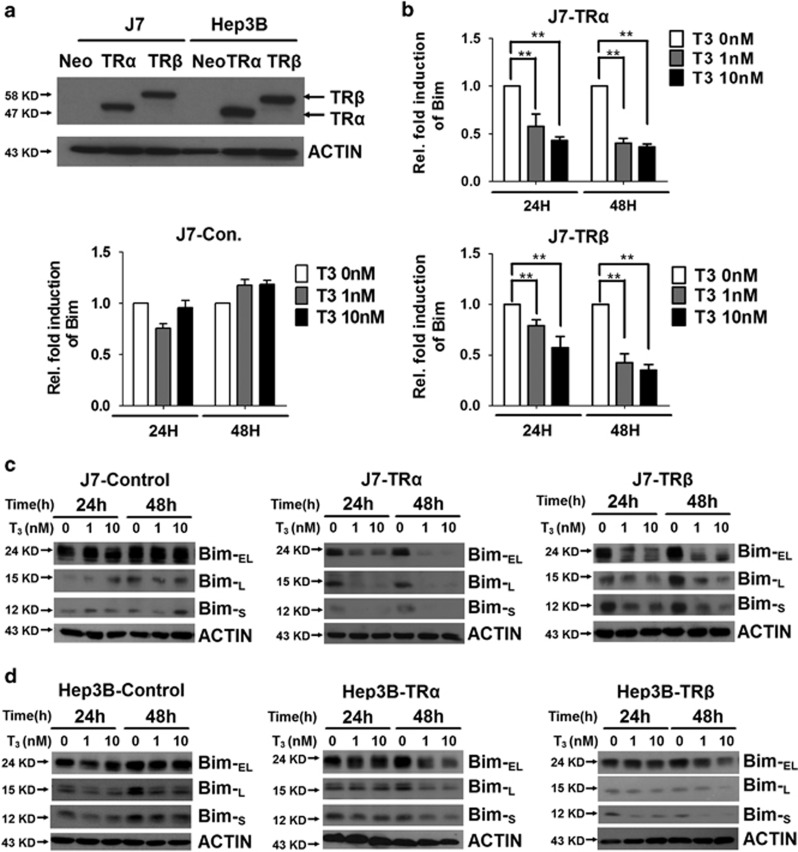
Effect of T_3_ on Bim mRNA and protein expression in hepatoma cell lines. (**a**) Detection of TR protein in TR-overexpressing or control J7 and Hep3B cell lines. (**b**) RNA from TR-overexpressing or control cell lines maintained in T_3_-depleted ([T_3_]=0 nM) or supplemented medium ([T_3_]=1 or 10 nM) for 24 or 48 h was prepared prior to qRT-PCR analysis of *Bim* mRNA. Values (means±S.E.M.) are shown as fold induction relative to 0 nM T_3_. All assays were repeated at least three times. **P<0.01; *P<0.05 (**c** and **d**) Levels of the three isoforms of Bim (Bim-_EL_, Bim-_L_ and Bim-_S_) in total lysates of isogenic J7 and Hep3B cell lines maintained in the absence or presence of T_3_ (1 or 10 nM) for 24 or 48 h were determined via western blotting. ACTIN signals served as the loading control

To explore the tumor-killing mechanism of TRs in HCC, the expression patterns of apoptosis-related genes in TR-overexpressing hepatoma cells following T_3_ treatment were examined via microarray. Among the range of apoptosis-related genes investigated, *Bim* was significantly downregulated upon T_3_ stimulation. To confirm this finding, ectopic expression of TR*α* and TR*β* with adenovirus in J7 and Hep3B hepatoma cell lines was performed (Figure 1a). The levels of *Bim* mRNA were suppressed in a time-, dose- and TR-dependent manner in various TR-overexpressing J7 cells following T_3_ treatment (Figure 1b). The effect of T_3_ on Bim protein expression was additionally assessed in isogenic cell lines derived from J7 or Hep3B. After incubation with 0 or 10 nM T_3_, levels of the three isoforms (EL, L and S) of Bim protein were dramatically suppressed in TR-overexpressing but not control cells (Figures 1c and d). Our results clearly indicate that T_3_ inhibits *Bim* expression at both the mRNA and protein level in a TR-dependent manner in hepatoma cells.

### T_3_/TR-dependent suppression of the *Bim* promoter is exerted via FoxO1

To determine whether T_3_/TR-mediated *Bim* downregulation occurs through transcriptional repression or other means, the human *Bim* promoter region encompassing nucleotides from upstream position −1995 to +338 (promoter region 1, P1) was cloned into pGL3-basic-TK vector, and regulation by TR was determined via measuring luciferase activity. Various deletion mutants from this region were additionally generated to pinpoint the crucial elements responsible for T_3_-induced *Bim* repression (promoter regions 15, P1–P5). Using these reporter constructs, the effects of transrepression of TR/T_3_ on the *Bim* 5′-flanking regions were determined.

**Figure 2:**
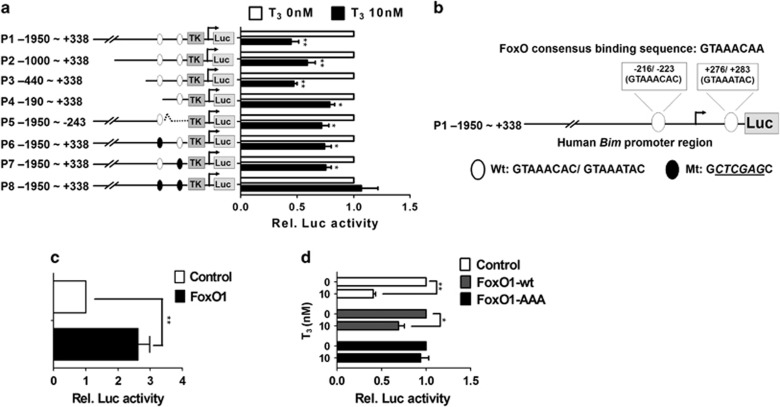
T_3_/TR-dependent suppression of the *Bim* promoter via FoxO1. (**a**) The *Bim* 5′-flanking region (−1950 to +338; P1), *Bim* 5′-deletion and FoxO1 consensus-binding site mutants (P1–P8) were cloned into pGL3-basic-TK vector to generate luciferase reporter plasmids. The promoter region in these mutants is shown (left). After co-transfection with *β*-galactosidase (a transfection efficiency control), J7-TR cells were harvested and luciferase activity was measured following treatment with T_3_ (0 or 10 nM) for 48 h. Luciferase activity was normalized to that of *β*-galactosidase (**P<0.01; *P<0.05). (**b**) The structure of the human *Bim* 5′-flanking region (positions −1950 to +338; P1) containing two putative FoxO1 consensus-binding sites. (**c**) Luciferase activities of the Bim promoter were analyzed in J7 cells after ectopic expression of control or FoxO1-wt via adenoviral transfection. Luciferase activity was normalized to that of *β*-galactosidase. Data represent means±S.E.M. of values derived from three independent experiments (**P<0.01; *P<0.05). (**d**) Following ectopic expression of control, FoxO1-wt or FoxO1-AAA via adenoviral (Ad-control, -FoxO1-wt or -FoxO1-AAA) infection in J7-TR cells, the effects of T_3_ on *Bim* promoter (positions −1950 to +338; P1) activity were examined as for panel (**a**)

Following the reporter activity assay, luciferase activities of the reporters driven by fragments −1995 to +338, −1000 to +338 and -440 to +338 (P1–P3) were dramatically suppressed in the presence of T_3_ in J7-TR cells. Interestingly, the suppressive effect of T_3_ on both −190 to +338 (P4) and −1950 to +243 (P5) regions was partially relieved. Previous studies have identified two putative FoxO1-binding elements, F-box1 (positions −216 to −223) and F-box 2 (positions +276 to +283), within this region that have critical roles in *Bim* activation.^22, 23^ We propose that these two F-boxes mediate the suppressive effect of T_3_ on the *Bim* promoter, in view of the finding that deletion of either one reduces the inhibitory effect of T_3_ (Figure 2b, P4 and P5). Site-directed mutagenesis of either F-box 1 (Figures 2a and b, P6) or F-box 2 (Figures 2a and b, P7) in the P1 promoter partially rescued T_3_-suppressed luciferase activity. Furthermore, mutation of both elements (P8) led to blockade of the inhibitory effect of T_3_ (Figure 2a). These data clearly suggest that F-box 1 and F-box 2 are the major *cis*-elements mediating the repressive effect of T_3_ on the *Bim* promoter.

To further establish whether T_3_-mediated transcriptional repression of *Bim* is FoxO1 dependent, we assessed the effect of FoxO1 on *Bim* promoter activity in J7 or J7-TR cells in the absence and presence of T_3_. *Bim* promoter-driven reporter assays showed that *FoxO1* overexpression enhances *Bim* promoter activity in J7 cells (Figure 2c). Additionally, ectopic overexpression of wild-type *FoxO1 (FoxO1-wt)* in J7-TR cells partially eliminated the effect of T_3_ on the *Bim* promoter (Figure 2c). Overexpression of *FoxO1-AAA*, a constitutively active form of FoxO1 (in which the three Akt phosphorylation sites are mutated to alanine) fully blocked the suppressive effect of T_3_ on the *Bim* promoter (Figure 2d). Our findings collectively demonstrate an important role of FoxO1 in T_3_-mediated regulation of *Bim* promoter activity and further suggest that T_3_ suppresses *Bim* promoter activity by interfering with FoxO1-mediated activation of the *Bim* promoter via regulating Akt-mediated phosphorylation of FoxO1.

### T_3_/TR represses *FoxO1* expression through genomic and non-genomic effects

**Figure 3:**
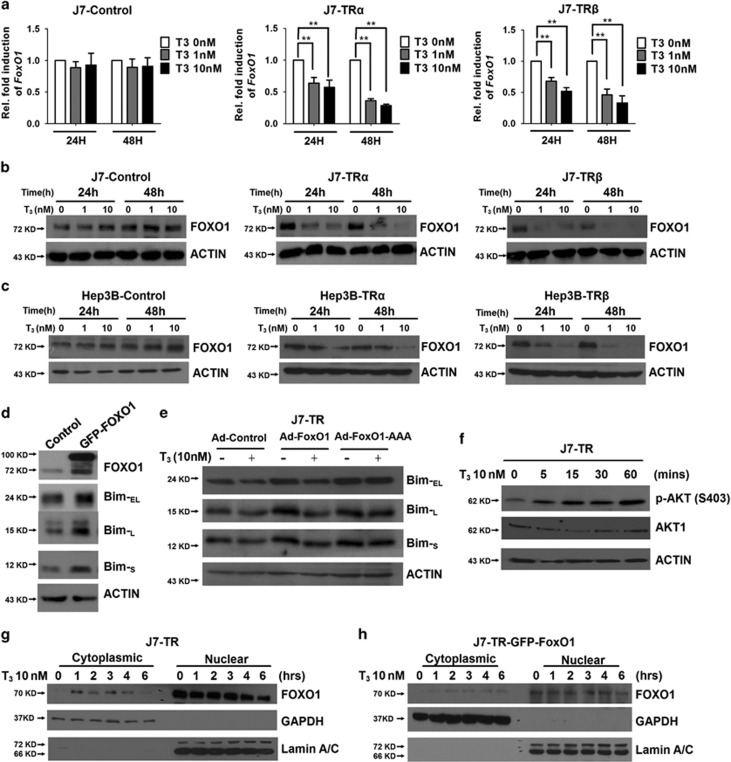
T_3_/TR represses Bim protein expression via downregulation of FoxO1. (**a**) RNA from TR-overexpressing or control J7 cells in the absence or presence of T_3_ for 24 or 48 h was prepared prior to qRT-PCR analysis of *FoxO1* mRNA expression. Values (means±S.E.M.) are shown as fold induction, compared with 0 nM T_3_. All assays were repeated at least three times (**P<0.01). (**b** and **c**) FoxO1 protein levels in total lysates of isogenic J7 and Hep3B cell lines maintained in the absence or presence of T_3_ (1 or 10 nM) for 24 or 48 h, determined using western blotting. ACTIN signals served as the loading control. (**d**) After transfection of GFP-FoxO1 for 48 h in J7 cells, the expression levels of FoxO1 and three isoforms of Bim were examined using western blotting. (**e**) Following ectopic expression of control, FoxO1-wt or FoxO1-AAA via adenoviral infection in J7-TR cells, the effects of T_3_ on Bim protein expression were examined using western blotting. (**f**) Following T_3_ (10 nM) stimulation for the indicated times, lysates of J7-TR cells were extracted for examining phosphor-AKT expression with western blotting. AKT1 and ACTIN were used as internal controls. (**g** and **h**) After T_3_ (10 nM) treatment for the indicated times, cytoplasmic and nuclear fractions of J7-TR or GFP-FoxO1-overexpressing J7-TR cells were extracted for detecting endogenous FoxO1 or exogenous GFP-FoxO1 protein expression

Although a crucial role of FoxO1 in T_3_-mediated transcriptional repression of *Bim* has been demonstrated, the issue of whether the FoxO1 transcription factor is regulated by T_3_/TR is yet to be clarified. RNA levels of *FoxO1* were dramatically repressed in J7 cells after incubation with T_3_ in a time-, dose- and TR-dependent manner (Figure 3a). Similarly, the protein levels of FoxO1 were repressed by T_3_ in TR-expressing J7 and Hep3B cell lines (Figures 3b and c). Notably, T_3_ effect on the repression of FoxO1 and Bim proteins was observed in Huh7, a hepatoma cell line expressing endogenous TR proteins. (Supplementary Figures S1A and B). Regulation of Bim expression by FoxO1 was examined by GFP-FoxO1 overexpression transiently. Dramatic enhancement of all isoforms of Bim protein was observed (Figure 3d). To further ascertain whether the suppressive effect of T_3_ on Bim protein occurs through FoxO1 downregulation, FoxO1-wt or FoxO1-AAA was overexpressed in J7-TR cells following T_3_ treatment. Interestingly, FoxO1-wt had only a minor effect in restoring the Bim protein level in the presence of T_3._ However, overexpression of *FoxO1-AAA*, diminished the T_3_-suppressive effect on Bim protein expression (Figure 3e). These data were consistent with the results of the reporter assays (Figure 2a) and suggest that T_3_ not only suppresses FoxO1 expression at the transcriptional level but also influences its activities via Akt-mediated nuclear exclusion or degradation. As T_3_/TR can trigger rapid, non-transcriptional effects through cross-coupling with the phosphatidylinositol 3-kinase (PI3K)-Akt pathway,^29^ we explored whether T_3_ influences FoxO1 activity in hepatoma cells through a non-genomic mechanism. The level of activated phospho-Akt was dynamically enhanced (Figure 3f), in parallel with downregulation of Bim and the nuclear exclusion of endogenous FoxO1 or exogenous GFP-FoxO1 after T_3_ treatment (Supplementary Figure S2 and Figures 3g and h). The results indicate that T_3_/TR represses FoxO1 through both genomic and non-genomic effects, leading to decreased *Bim* expression.

### Bim acts synergistically with cisplatin, doxorubicin and tumor necrosis factor-related apoptosis-inducing ligand (TRAIL) to kill hepatoma cells

**Figure 4:**
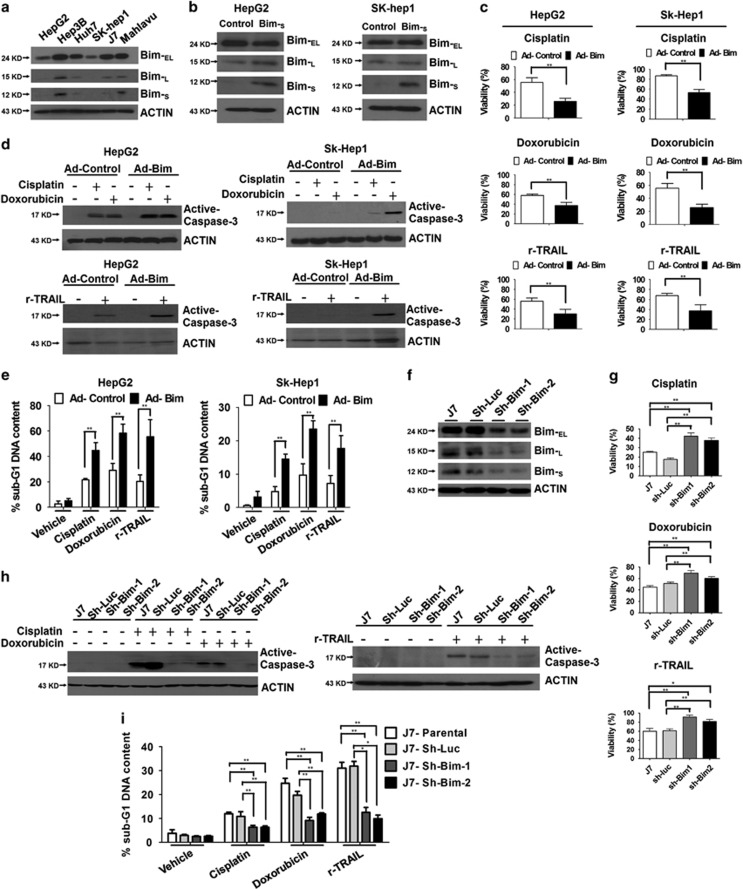
Bim acts synergistically with cisplatin, doxorubicin and TRAIL to kill hepatoma cells. Detection of Bim proteins in (**a**) parental HCC cell lines and (**b**) Ad-Bim-infected HepG2 or SK-hep1 cells. (**c**) Ad-control or Ad-Bim infected cells were treated with cisplatin (2.5 *μ*g/ml), doxorubicin (2.5 *μ*M) or r-TRAIL (10 ng/ml) for 48 h. After stimulation, cell viability was measured using the MTT assay, and data are presented as relative absorbance values (%) of vehicle-treated cells. (**d** and **e**) Control and Bim-overexpressing cells were treated with the indicated reagents for 24 h, the extent of caspase-3 activation determined via western blotting (**d**), and the proportions of apoptotic cells in samples subjected to various treatments assessed using propidium iodide (PI) staining and flow cytometry (**e**). Values are presented as means±S.E.M. of data from experiments performed in triplicate. Differences between data were evaluated using Student's *t*-test (**P<0.01; *P<0.05). (**f**) Immunoblot analysis of the expression patterns of Bim proteins in J7 cells infected with lentivirus expressing luciferase (Sh-Luc) or Bim-targeting *shRNA*. (**g**) Control (Sh-Luc) or Bim-depleted (Sh-Bim) cells were treated with cisplatin (2.5 *μ*g/ml), doxorubicin (2.5 *μ*M) or r-TRAIL (10 ng/ml) for 48 h, and cell viabilities were determined via the MTT assay. The extent of caspase-3 activation and proportion of apoptotic cells were determined via (**h**) western blotting and (**i**) flow cytometry, respectively, and cell viability was measured using the MTT assay. (**h**) After treatment with the indicated reagents for 24 h, caspase-3 activation in control or Bim-depleted cells was determined via western blotting analysis. (**i**) PI staining and flow cytometry were performed to assess the proportion of the sub-G1 phase in samples subjected to various treatments

The expression levels of Bim proteins in several hepatoma cells with various invasion/metastatic capabilities at distinct differentiation stages were compared. Bim proteins were highly enriched in Hep3B and Mahlavu but present at lower levels in HepG2 and SK-Hep1 cells (Figure 4a). To ascertain whether higher levels of Bim sensitize hepatoma cells to chemotherapeutic drugs or tumor-killing cytokines, Bim-_S_ was overexpressed via adenoviral transduction in HepG2 and Sk-Hep1 cell lines displaying low Bim expression, followed by challenge with the anticancer drugs cisplatin, doxorubicin or recombinant (r)-TRAIL (Figure 4b). Cell viability results with both cell types indicated enhanced cisplatin, doxorubicin and r-TRAIL-induced cell death in *Bim-*s-overexpressing cells (Figure 4c). Moreover, although all the tested drugs triggered apoptotic events, as evident from increased activation of Caspase-3 and the number of cells in the sub-G1 populations, their effects were significantly enhanced upon Bim overexpression in HepG2 and Sk-Hep1 cells (Figures 4d and e). To further determine the influence of Bim on anticancer drug-mediated apoptosis of hepatoma cells, expression in J7, the hepatoma cell line containing physiologically high levels of *Bim*, was reduced using the *shRNA*-mediated knockdown approach (Figure 4f). As expected, the extent of cisplatin, doxorubicin or r-TRAIL-induced apoptosis (indicated by cell viability and active caspase 3 levels) was dramatically reduced in *Bim*-depleted J7 cells (Figures 4g–i). These results suggest that Bim acts as a synergistic factor to induce apoptosis in hepatoma cells challenged with chemotherapeutic drugs or tumor-killing cytokines, such as TRAIL.

### T_3_-mediated *Bim* downregulation protects hepatoma cells against cisplatin, doxorubicin and TRAIL-induced apoptosis

**Figure 5:**
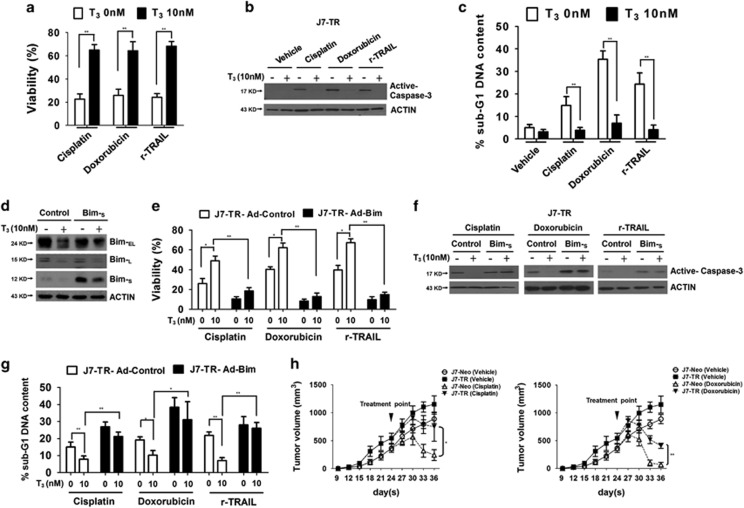
T_3_/TR-induced cisplatin, doxorubicin and TRAIL resistance is mediated via Bim downregulation. (**a**) J7-TR cells were treated with the indicated reagents for 24 h after T_3_ (0 or 10 nM) stimulation for 48 h, and cell viability was measured using the MTT assay. Data are presented as relative absorbance values (%) of vehicle-treated cells. The extent of caspase-3 activation and proportion of apoptotic cells were determined via (**b**) western blotting and (**c**) flow cytometry, respectively. (**d**) Bim-_S_ was ectopically overexpressed in J7-TR cells via adenovirus infection in the presence or absence of T_3_ for 48 h, and Bim proteins were determined via western blotting. Cells were subsequently stimulated with the indicated agents for 24 h. (**e**) Cell viabilities were determined with the MTT assay. (**f** and **g**) Caspase-3 activation was determined via western blotting and the percentage of apoptotic cells was assessed with PI-stained flow cytometry. (**h**) J7-Neo and J7-TR cells were subcutaneously injected into the flanks of nude mice. At 24 days after tumor inoculation, mice were treated with vehicle, cisplatin or doxorubicin. Tumor volumes were measured once every 3 days, and tumor growth curves are shown. Differences between data were evaluated using Student's *t*-test (**P<0.01; *P<0.05)

To further clarify the influence of T_3_/TR on activities of anticancer drugs, TR-overexpressing J7 cells and Huh7 cells were stimulated with several chemotherapeutic drugs and r-TRAIL. The apoptotic events following addition of cisplatin, doxorubicin and r-TRAIL were significantly reduced in T_3_-pretreated J7-TR and Huh7 cells (Figures 5a–c and Supplementary Figure S3). We were interested in clarifying whether T_3_ influences the sensitivity of hepatoma cells to these drugs via downregulation of *Bim*. To this end, *Bim-*_*S*_ was ectopically expressed in J7-TR and Huh7 cells with or without T_3_ treatment (Figure 5d and Supplementary Figure S4A). Interestingly, although T_3_ pretreatment protected J7-TR and Huh7 cells control cells from apoptosis induced by chemotherapeutic agents, the antiapoptotic effect of T_3_ was abolished in *Bim*-overexpressing cells (Figures 5e–g and Supplementary Figures 4B–D). The protective effect of T_3_/TR against drug-induced cell death was further studied *in vivo* using subcutaneous xenografts of control (J7-Neo) and TR-overexpressing J7 cells (J7-TR) in euthyroid nude mice. Concordant with *in vitro* results, TR overexpression conferred not only tumor growth advantage but also resistance to cisplatin and doxorubicin in J7 hepatoma cells (Figure 5h). Based on these collective findings, we propose that T_3_/TR inhibits the apoptotic effects of chemotherapeutic drugs in hepatoma cells through suppressing expression of the apoptosis regulator *FoxO1* and its downstream target *Bim*.

### Bim suppresses TH/TR-enhanced doxorubicin resistance and metastasis

**Figure 6:**
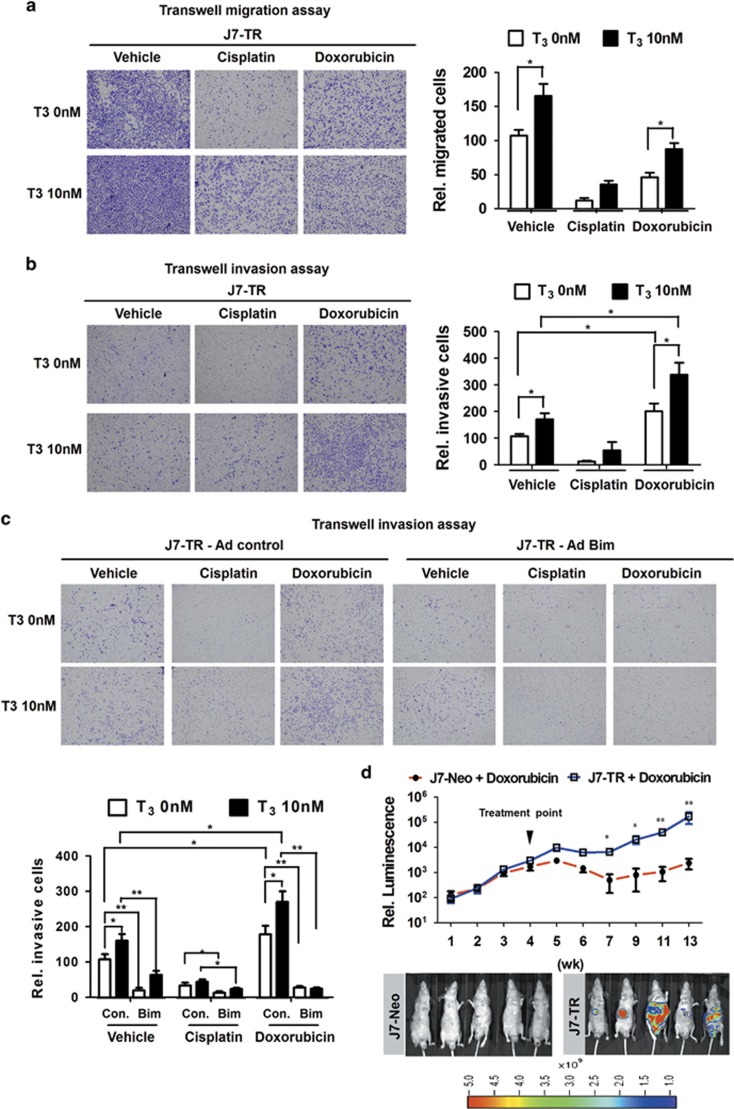
Bim suppresses TH/TR-enhanced doxorubicin resistance and metastasis. (**a**) In the presence of cisplatin (2.5 *μ*g/ml) and doxorubicin (2.5 *μ*M), the migratory and (**b**) invasive properties of J7-TR cells treated with or without T_3_ were assessed using the transwell assay. Images of traversing cells are shown in the left panels and the quantitative results in the right panels. To assess the influence of Bim on chemotherapeutic drugs-mediated invasion of J7-TR cells, (**c**) invasive properties of control or Bim-overexpressing J7-TR cells were stimulated with cisplatin or doxorubicin in the absence or presence of T_3_ and subsequently assessed using the transwell assay. Images of traversing cells are shown in the upper panels and the quantitative results in the lower panels. Differences between data were evaluated using Student's *t*-test (**P<0.01; *P<0.05). (**d**) Tumors dissected from mice were subjected to subcutaneous injection with Luc-GFP-expressing J7-Neo and J7-TR cells were introduced orthotopically into the livers of nude mice and monitored weekly with IVIS. After 4 weeks, mice were treated with vehicle or doxorubicin (10 mg/kg, once a week). Relative intensity of luminescence from IVIS is shown in the upper panel, and representative IVIS images 13 weeks after tumor inoculation are shown in the lower panels

Several lines of evidence suggest that chemotherapeutic drugs alternatively have the potential to accelerate tumor progression of apoptosis-resistant cells.^30^ To further explore this issue, a transwell assay was employed to determine the metastatic capabilities of T_3_-treated J7-TR cells in the presence of cisplatin or doxorubicin. cisplatin treatment dramatically inhibited cellular migration and invasion, regardless of T_3_ (Figures 6a and b). Interestingly, doxorubicin suppressed cellular migration but enhanced the invasive potential of J7-TR cells. Additionally, T_3_ treatment further accelerated doxorubicin-mediated cellular invasion (Figure 6b). The results imply that T_3_ stimulates apoptosis resistance of J7-TR cells and, subsequently, doxorubicin-enhanced invasion. To ascertain whether Bim has the ability to eliminate the effects of T_3_ and doxorubicin on cellular invasion, ectopic expression of Bim in J7-TR and Huh7 cells was achieved, followed by transwell invasion analysis. Notably, Bim expression led to dramatic suppression of the invasive ability of J7-TR and Huh7 cells, even in the presence of doxorubicin and T_3_ (Figure 6c and Supplementary Figure S5). To further ascertain whether these T_3_/TR-associated effects are replicated *in vivo*, the orthotopic mouse model was employed. Tumor growth and metastasis were monitored with *in vivo* imaging system (IVIS) weekly after liver implantation of Luc-GFP-expressing J7-Neo or J7-TR tumors. Concordant with the *in vitro* results, TR overexpression not only caused tumor resistance to doxorubicin but also increased the metastatic potential of J7 hepatoma cells *in vivo* (Figure 6d). Taken together, these observations confirm that T_3_/TR inhibits the apoptotic effects of doxorubicin through Bim downregulation, subsequently promoting tumor progression.

## Discussion

HCC is the fifth most common cancer and the third most common cause of cancer-related mortality worldwide. More than 748 000 newly diagnosed cases and about 700 000 deaths occur annually.^7, 31^ Because of the advanced stage of this disease at the time of diagnosis, <30–40% of HCC cases are eligible for curative treatments, including surgery, liver transplantation or percutaneous ablation.^32^ Eventually, the majority of HCC patients require chemotherapy. To date, doxorubicin has been widely used as a chemotherapeutic drug for advanced HCC but displays low efficacy with a ~15–20% response rate.^32^ Other chemotherapeutic agents, such as cisplatin, Etoposide, 5-Fluorouracil and their combinations, demonstrate even lower efficacy.^33^ Because of the poor response to the currently available chemotherapeutic agents, continuous efforts have been made to establish new molecular targets or signaling pathways for developing more effective drugs.

Circulating TH and its cognate receptors have pivotal roles in controlling cellular development and metabolic homeostasis in vertebrates. Additionally, it is increasingly apparent that TH/TRs have important roles in HCC development, with several documented roles in tumorigenesis. For instance, expression levels of TRs and their target genes are decreased in early preneoplastic lesions and hepatocellular HCC in rats, and local hypothyroid status may favor the onset and progression of preneoplastic lesions to HCC.^34^ A recent epidemiological study also suggested that long-term hypothyroidism is positively correlated with HCC incidence, independent of other risk factors.^35^ However, the proposed tumor-suppressor role of TRs remains a subject of controversy. For example, an animal model of colorectal tumorigenesis revealed that TR*α*1 positively regulates several molecules downstream of the Wnt signal, consequently facilitating the expression of *β*-catenin/Tcf4 target genes and enhancing cell growth. Intriguingly, in a strain of Wnt-activated Apc^+^/^1638N^ mice, TR*α* overexpression in the intestinal epithelium did not induce cancer formation but rather accelerated tumorigenesis.^36^ Recently, another group reported that TRs act as potent suppressors of tumor metastasis in breast cancer cell lines.^37^ The investigators further showed that mice with double knockout of *TRα* and *TRβ* are vulnerable to epithelial tumors and that TR deficiency suppresses the number of benign tumors but enhances malignant tumor formation during carcinogenesis. Thus TH/TR appears to perform dual roles, mitogenic and tumor suppressive, in various tissues, although the mechanisms by which these two functions are coupled remain to be elucidated.

Adult liver is a quiescent organ that exhibits an extremely low mitosis frequency (<1/20 000 hepatocytes). However, several lines of evidence suggest that liver damage caused by partial hepatectomy or other means (including chemical-, nutrition-, vascular- or viral-mediated liver injury) strongly enhances the hepatocyte turnover rate to regenerate damaged parts. These stimulated hepatocytes have higher potential to generate tumor precursor cells that later develop into preneoplastic lesions or HCC.^38, 39, 40^ Previous studies using the DEN-induced HCC animal model have suggested that T_3_ serves as a hepatomitogenic factor to stimulate liver cell growth but without associated cell death, consequently leading to decreased preneoplastic hepatic lesions.^10, 11^ However, in advanced HCC cells, the TH may exert a pro-survival function, causing chemotherapeutic drug resistance. The mitogenic effect of TH may promote malignant hepatic tumor progression or chemoresistance but requires further investigation. In the current study, we confirmed the protective role of T_3_/TR in hepatoma cells following challenge with several chemotherapeutic agents. Our data suggest that this function is attributable to downregulation of *FoxO1* through both transcriptional repression and Akt-mediated nuclear exclusion, subsequently leading to transcriptional repression of *Bim.*

The FoxO transcription factors are important regulators of cell cycle arrest and apoptosis acting downstream of PTEN.^41^ These proteins act as important tumor suppressors as they induce cell cycle arrest at the G1–S checkpoint through activation of *p27*^*Kip1*^ or the G2–M checkpoint via activation of *GAD45* to stimulate the DNA repair pathway.^41, 42, 43^ Furthermore, under sustained stress conditions, FoxOs induce expression of the pro-apoptosis gene, *Bim*, to trigger programmed death of overstressed/damaged cells.^22, 23^ The ability of FoxO proteins to induce cell arrest and death at multiple steps has attracted considerable research efforts to assess their roles in carcinogenesis or as targets in cancer therapy. A number of lines of evidence have shown that Bim is the major mediator of the cell death effect of various anticancer drugs, including cisplatin and doxorubicin, and its degradation/downregulation is responsible for resistance to these drugs. Therefore, induction of Bim by FoxO may have a key role in maintaining the Bim level in cancer cells and dictating their response to drugs. The current discovery that T_3_/TR can mitigate the FoxO/Bim cascade in HCC cells suggests that the TR expression level has a significant effect on patient responses to therapeutic drugs. Thus manipulating the T_3_/TR level in patients may become an important issue in cancer therapy.

Acquisition of anchorage independence is a crucial step for tumor metastasis. Cell life is dependent on anchorage, and cells undergo apoptosis after loss of attachment to ECM or adherence with their neighboring cells. Thus apoptosis induced by cell detachment, designated anoikis, is a critical barrier against tumor metastasis. Bim has a key role in anoikis in several cancer types, including breast cancer, lung cancer, osteosarcoma and melanoma.^44, 45, 46^ In these cancer types, abrogation of Bim-mediated cell death is required for metastasis. Our data indicate that doxorubicin has the potential to induce hepatoma cell invasion. Notably, T_3_-induced *Bim* repression not only caused chemotherapeutic drug resistance but also enhanced doxorubicin-induced metastasis of hepatoma cells. Conversely, ectopic expression of *Bim* in hepatoma cells strongly increased sensitivity to chemotherapeutic drugs and suppressed doxorubicin-induced metastasis, consequently abolishing TH/TR-enhanced apoptosis resistance and metastasis following drug treatment. The clinical relevance of the TH/TR-suppressed FoxO/Bim cascade needs to be further investigated to establish whether patients with abnormal levels (high or low) of TR show different degrees of metastasis.

Doxorubicin is one of the most commonly used chemotherapeutic drugs. However, several lines of evidence, including data from the current study, support its potential to accelerate malignant tumor progression, with a number of mechanisms proposed to explain this phenomenon. For instance, doxorubicin induces epithelial–mesenchymal transition and cell migration through TGF*β* signaling activation in breast cancer.^30^ MMP-2 and -9, the crucial enzymes that degrade the ECM to facilitate tumor invasion, are activated by doxorubicin.^47^ Although the current findings suggest that suppression of *Bim* expression accounts to a large extent for TH/TR-enhanced doxorubicin-mediated metastasis, further studies are essential to ascertain whether metastasis is also enhanced via the above mechanisms. Our observations provide conclusive molecular evidence supporting targeting of the TH and Bim in chemotherapy regimens for HCC.

## Materials and Methods

### Cell culture

Hepatoma cell lines, including HepG2, Sk-Hep1, Hep3B and isogenic J7, were routinely cultured in Dulbecco's Modified Eagle's Medium (DMEM) supplemented with 10% FBS (v/v) at 37 °C in a humidified atmosphere containing 5% (v/v) CO_2_ and 95% (v/v) air. T_3_-depleted (T_3_, 0 nM) serum was prepared by treatment with AG 1-X8 resin (Bio-Rad, Hercules, CA, USA) and added to DMEM at 10% (v/v) to form Td medium. T_3_ was purchased from Sigma-Aldrich (St. Louis, MO, USA).

### Real-time PCR (qRT-PCR)

Total RNA of hepatoma cells was extracted using TRIzol reagent (Life Technologies Inc., Carlsbad, CA, USA), and cDNA strands were synthesized using the Superscript III Kit for RT-PCR (Life Technologies). Real-time quantitative RT-PCR was performed using SYBR Green reaction mix (Applied Biosystems, Carlsbad, CA, USA), and products detected using the ABI PRISM 7500 system (Applied Biosystems, Foster City, CA, USA).

### Western blotting

Cell extracts were fractionated via SDS-PAGE on a 12% gel, and the separated proteins were transferred to a polyvinylidene fluoride membrane. Subsequent procedures were performed as described previously.^48^ Rabbit anti-human antibodies against Bim and active caspase-3 were purchased from Epitomics (Burlingame, CA, USA). The rabbit anti-GFP antibody was obtained from GeneTex (Irvine, CA, USA). Rabbit antibodies against FoxO1 were acquired from Cell Signaling Technology (Boston, MA, USA).

### Transient transfection and reporter assays

The 5′-flanking region (positions −1950 to +338) of the *Bim* gene was amplified and cloned into pGL3-TK vector to generate a P1 reporter plasmid. Sequential 5′or 3′-deletions (P2–P5) and mutants of the FoxO1 consensus-binding element (P6–P8) derived from this reporter were generated. To explore *Bim* promoter response to T_3_ or FoxO1 stimulation, J7-TR, J7-FoxO1-WT or J7-FoxO1-AAA cells were transfected with 0.2 μg of *Bim* promoter-driven reporter constructs and 0.05 *μ*g of SV*β* vector expressing *β*-galactosidase (Clontech, Palo Alto, CA, USA) for 16 h. Cells were subsequently treated with T_3_ (0 or 10 nM) and incubated for 48 h prior to harvesting. Activities of luciferase and *β*-galactosidase were subsequently measured.

### *In vitro* invasion assay

The influence of Bim on doxorubicin or T_3_/TR-mediated invasive activity of TR-overexpressing hepatoma cell lines was assessed using the rapid Transwell *in vitro* assay. After adjusting cell density to 1 × 10^5^ cells/100 *μ*l of serum-free DMEM, cells were added to each upper chamber coated with Matrigel (Becton-Dickinson, Franklin Lakes, NJ, USA). The lower chamber contained DMEM supplemented with 20% (v/v) FBS. Following incubation for 16 h at 37 °C, cells traversing the filter to the lower chamber were stained with crystal violet and counted. All assays were repeated at least three times. Among-treatment differences were explored using one-way ANOVA (*P<0.05; **P<0.01).

### Xenograft mouse model

J7-Neo and J7-TR cells (1 × 10^6^) were subcutaneously injected into the flanks of nude mice (BALB/cAnN.Cg-Foxn1^nu^/CrlNarl). At 24 days after tumor inoculation, mice were treated with vehicle, cisplatin (10 mg/kg once every 3 days, i.p) or doxorubicin (10 mg/kg once every 3 days, i.p). Tumor volumes were measured twice a week (*n*=4 for each group), and tumor growth curves are shown.

### Orthotopic mouse model

Tissues of subcutaneously grown Luc-GFP-expressing J7-Neo and J7-TR hepatoma cells were sectioned into 1 mm^3^ pieces. Tumor sections were transplanted into livers of nude mice, as described earlier.^49^ After 4 weeks, mice were treated with vehicle or doxorubicin (10 mg/kg once per week, i.p). Cellular growth and metastasis of tumors were monitored once a week via IVIS.^50^ After 13 weeks, mice were killed, and the livers and lungs are collected.

### Flow cytometric assay of apoptosis

Parental or derived hepatoma cells were treated with cisplatin, doxorubicin or r-TRAIL after stimulation with T_3_ (0 or 10 nM) for 24 h. Cells were harvested via trypsinization and fixed in ethanol/PBS (7:3, v/v) for 1 h at −20 °C. Subsequently, cells were washed with PBS and resuspended in PBS containing 40 *μ*g/ml RNase A and 0.5% (v/v) Triton X-100 for 1 h at 37 °C. Finally, cells were pelleted and resuspended in PBS containing propidium iodide (50 μg/ml, Sigma). The extent of genomic DNA fragmentation was quantified via flow cytometric analysis of hypodiploid DNA. Data were collected and analyzed with FACScan (Becton Dickinson, San Jose, CA, USA) running the CellQuest software (Becton Dickinson, San Jose, CA, USA).

## Abbreviations

BH3-only: Bcl-2-homology domain 3 only
Bim: Bcl-2-interacting mediator of cell death
DEN: diethylnitrosamine
ECM: extracellular matrix
FoxO: transcription factors of the class O
HCC: hepatocellular carcinoma
MMP: matrix metalloproteinase
PI3K: phosphatidylinositol 3-kinase
TH: thyroid hormone
TR: thyroid hormone receptor
TRAIL: tumor necrosis factor-related apoptosis-inducing ligand
