# Supplementary material for: Chemotherapy resistance and metastasis-promoting effects of thyroid hormone in hepatocarcinoma cells are mediated by suppression of FoxO1 and Bim pathway
Source: Cell Death Dis. 2016 Aug 4;7(8):e2324–. doi: 10.1038/cddis.2016.227 (PMC5108316; doi:10.1038/cddis.2016.227)
Supplement: Supplementary Figures [file cddis2016227x1.pdf]

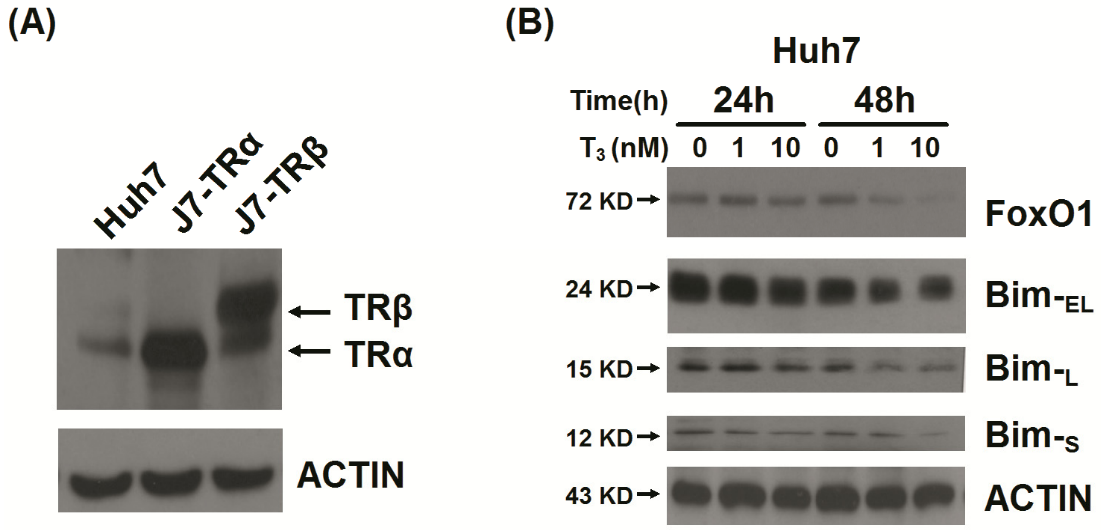

Supplementary Figure 1. Effect of T_3 on protein expressions of FoxO1 and Bim in Huh7 cells

(A) Detection of TR protein in Huh7 and TRs-overexpressing J7 cells. (B) Expression levels of FoxO1 and three isoforms of Bim in total lysates of Huh7 maintained in the absence or presence of T_3 (1 or 10 nM) for 24 or 48 h were determined via western blot. ACTIN signals served as the loading control.

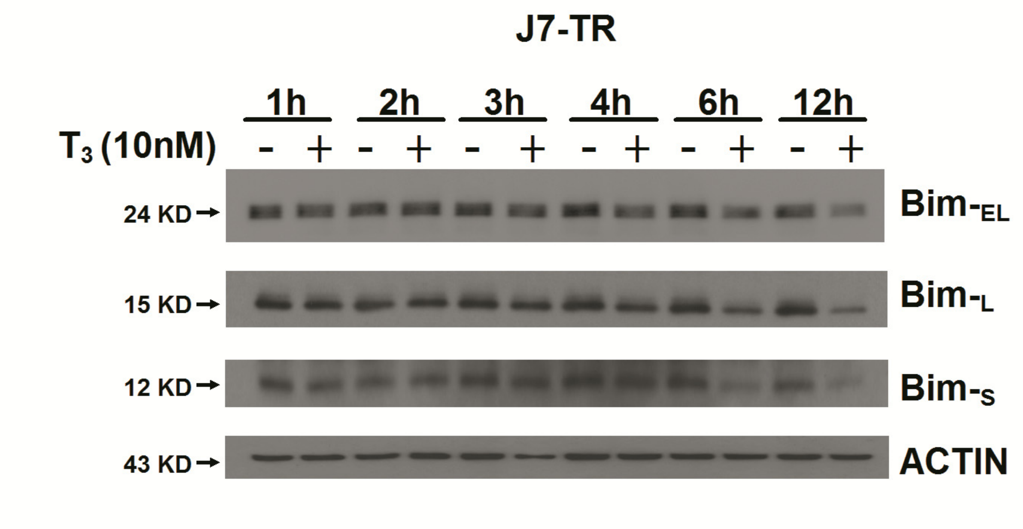

Supplementary Figure 2. T_3/TR represses Bim protein expression in J7 cells
Following T_3 (10 nM) stimulation for the indicated times, lysates of J7-TR cells were extracted for examining Bim proteins with western blot.

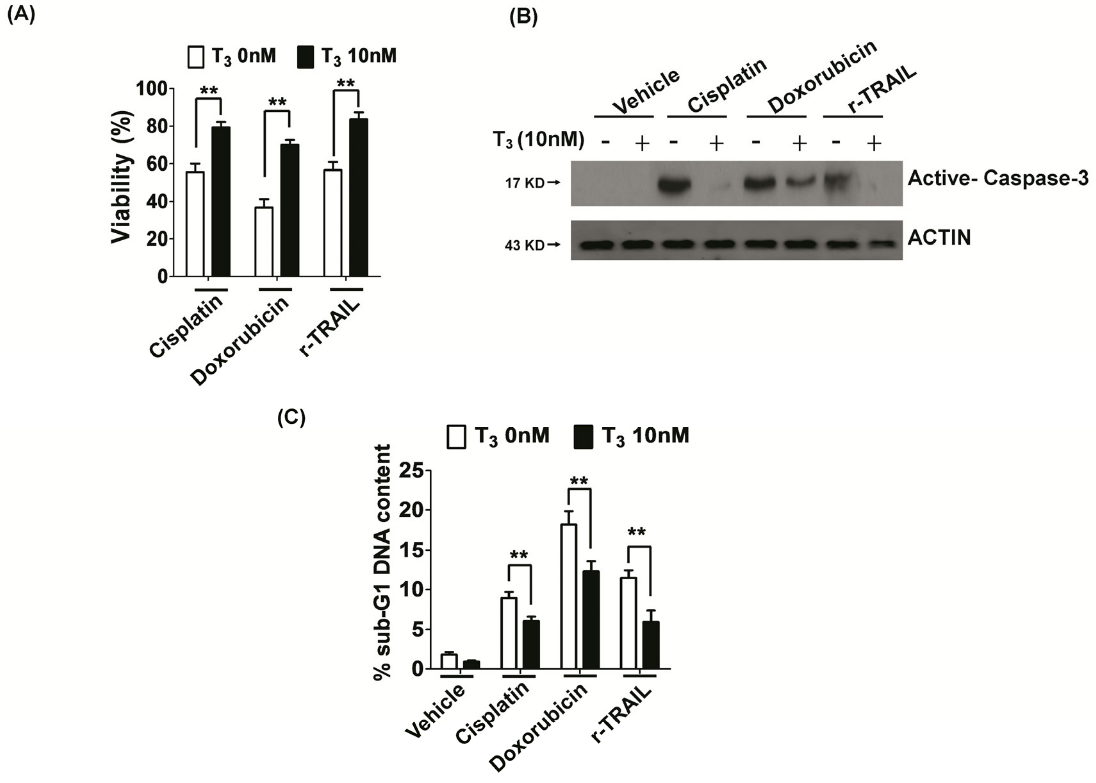

Supplementary Figure 3. T_3 induced cisplatin, Doxorubicin and TRAIL resistance in Huh7 cells

(A) Huh7 cells were treated with the indicated reagents for 24 h after T_3 (0 or 10 nM) stimulation for 48 h, and cell viability measured using the MTT assay. Data are presented as relative absorbance values (%) of vehicle-treated cells. The extent of caspase-3 activation and proportion of apoptotic cells were determined via western blot (B) and flow cytometry (C), respectively.

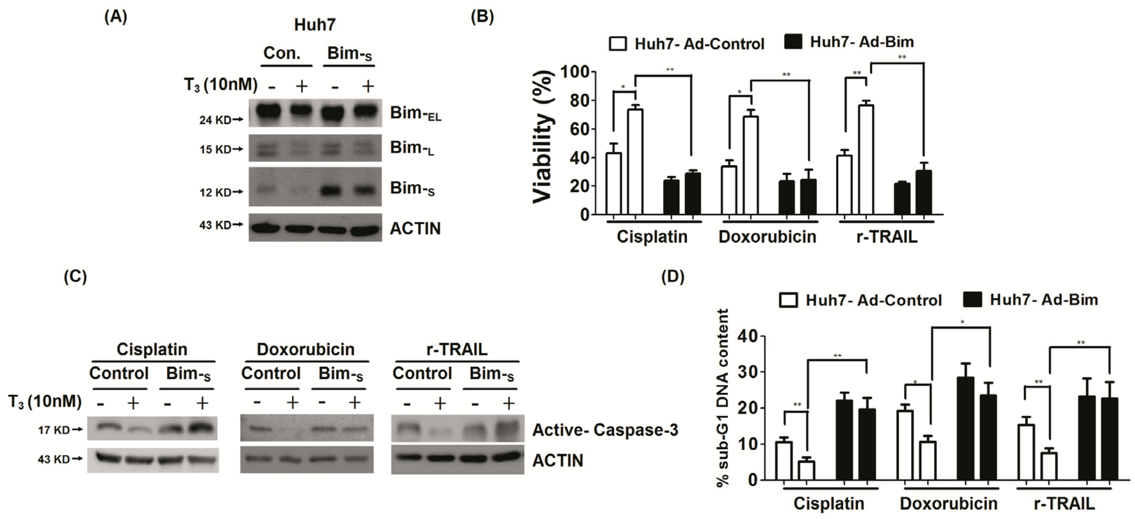

Supplementary Figure 4. T_3 induced cisplatin, Doxorubicin and TRAIL resistance is mediated via Bim downregulation in Huh7 cells

(A) Bim-$_S$ was ectopically overexpressed in Huh7 cells via adenovirus infection in the presence or absence of T_3 for 48 h, and Bim proteins determined via western blot. Cells were subsequently stimulated with the indicated agents for 24 h. (B) Cell viabilities were determined by the MTT assay. (C, D) Caspase-3 activation was determined via western blot and the percentage of apoptotic cells assessed with PI-stained flow cytometry.

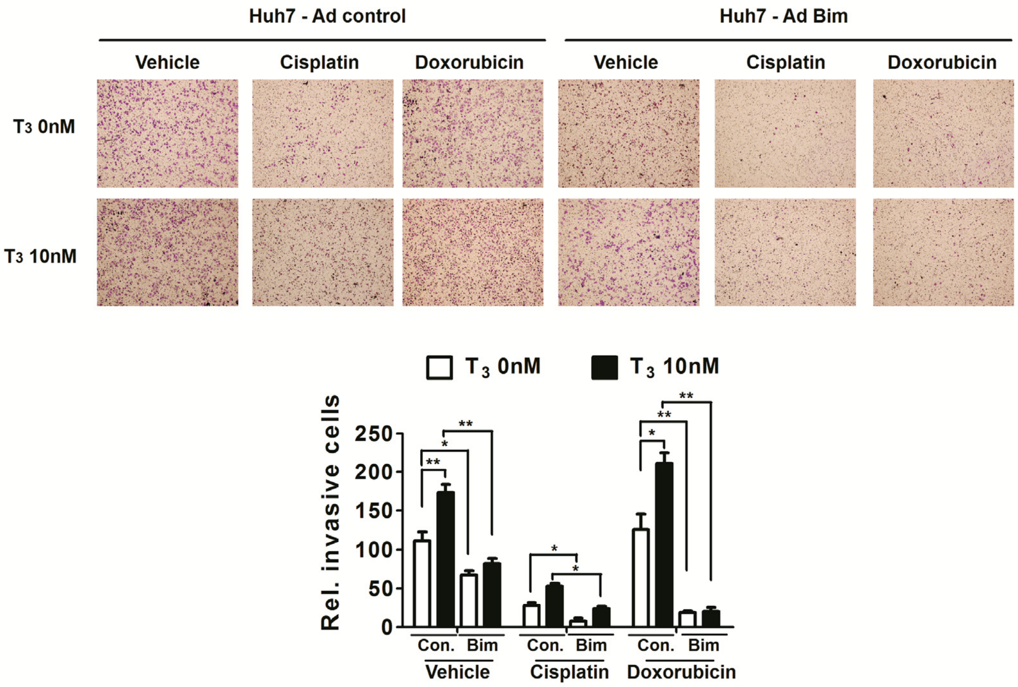

Supplementary Figure 5. Bim suppresses T_3 enhanced doxorubicin resistance and metastasis in Huh7 cells

(A) In the presence of cisplatin (2.5 μg/ml) and doxorubicin (2.5 μM), the invasive properties of Huh7 cells treated with or without T_3 were assessed using the transwell assay. Images of traversing cells are shown in the left panels, and the quantitative results in the lower panels.
